# Electrochemical Sensing toward Trace As(III) Based on Mesoporous MnFe_2_O_4_/Au Hybrid Nanospheres Modified Glass Carbon Electrode

**DOI:** 10.3390/s16060935

**Published:** 2016-06-22

**Authors:** Shaofeng Zhou, Xiaojuan Han, Honglei Fan, Yaqing Liu

**Affiliations:** 1 Shanxi Province Key Laboratory of Functional Nanocomposites, North University of China, Taiyuan 030051, China; hanxj2015nn@163.com; 2 Shanxi Province Key Laboratory of Higee-Oriented Chemical Engineering, North University of China, Taiyuan 030051, China; whutfanfan@163.com

**Keywords:** arsenite, MnFe_2_O_4_/Au, electrochemical detection, SWASV

## Abstract

Au nanoparticles decorated mesoporous MnFe_2_O_4_ nanocrystal clusters (MnFe_2_O_4_/Au hybrid nanospheres) were used for the electrochemical sensing of As(III) by square wave anodic stripping voltammetry (SWASV). Modified on a cheap glass carbon electrode, these MnFe_2_O_4_/Au hybrid nanospheres show favorable sensitivity (0.315 μA/ppb) and limit of detection (LOD) (3.37 ppb) toward As(III) under the optimized conditions in 0.1 M NaAc-HAc (pH 5.0) by depositing for 150 s at the deposition potential of −0.9 V. No obvious interference from Cd(II) and Hg(II) was recognized during the detection of As(III). Additionally, the developed electrode displayed good reproducibility, stability, and repeatability, and offered potential practical applicability for electrochemical detection of As(III) in real water samples. The present work provides a potential method for the design of new and cheap sensors in the application of electrochemical determination toward trace As(III) and other toxic metal ions.

## 1. Introduction

Inorganic arsenic contamination in drinking water has become a serious worldwide threat to human health due to arsenic’s high toxicity [1]. Such pollutants in drinking water may lead to many health problems, such as skin lesions, keratosis, lung cancer, and bladder cancer [2]. According to reports for twenty countries, the arsenic levels in drinking water is higher than the World Health Organization (WHO)’s arsenic guideline value of 10 μg·L^−1^ (*i.e*., 10 ppb) [3,4]. Thus, it is rather important to have an accurate, rapid, and sensitive method to detect and monitor the environmental pollution of drinking water. Although various spectroscopic methods are excellent for arsenic testing in labs, they are very expensive and not suitable for *in situ* analyses due to the lengthy and complex instruments required [5,6]. However, electrochemical methods, particularly stripping voltammetry analysis, have provided promising techniques that are available for the sensitive detection and quantification of arsenic due to their low cost, portability, and suitability for on-site analysis [7,8,9]. The electrochemical application and performance of an electrode depends to a large extent on the materials from which it has been made. For the electrochemical detection of arsenic, a large effort has been poured into electrode materials and the modification of electrode surfaces in an attempt to improve their analytical performance [7,10]. So far, Au has been shown to be a promising choice in the sensitive determination of arsenic due to its excellent electrocatalytic ability [11,12]. However, considering the potential obstacles associated with using a solid gold macroelectrode, such as high costs, rigorous control, and care of the quality of its surface, Au nanoparticle modified electrodes have drawn extensive attention and bring a new area to the voltammetric detection of inorganic arsenic [3,13]. It was reported that the Au nanoparticle modified electrode can behave as random arrays of microelectrodes with the superiority of enhanced mass transfer, efficient catalysis, and a controllable microenvironment [14,15,16].

Recently, metal-oxide nanomaterials have been creatively introduced into voltammetric responses for inorganic arsenic due to their excellent catalytic activity and adsorption ability [17,18,19]. Fe_3_O_4_, as a low-cost, eco-friendly, and easily-prepared material with ideal catalytic and absorption activity, has attracted great attention for its high sensitivity and rapid response toward arsenic via the electrochemical method [7]. Gao reported using Fe_3_O_4_ microspheres composited with room-temperature ionic liquids (RTILs) for the determination of As(III), which reached an exciting electrochemical performance (sensitivity: 4.91 μA/ppb; LOD: 0.0008 ppb) for the detection of As(III) through square wave anodic stripping voltammetry (SWASV) compared with the noble-metal nanoparticle modified electrodes [18]. Monodispersed Fe_3_O_4_ nanocrystals were also reported that could be exploited in order to remove arsenic from water due to their strong adsorption capacity [20]. To enhance the electrochemical behavior of Fe oxides toward arsenic, it was found that the addition of another metallic element not only changed the surface characteristics and micro-structure of the Fe oxide, but also participated in the process of arsenic removal [21]. Noteworthy, MnO_2_ plays a role as an oxidant in the As(III) uptake by FMBO and MNFHO, which could perform much better than pure Fe oxides for the removal of As(III). [22,23]. Xu *et al.* reported the MnFe_2_O_4_ combined with uniform mesoporous structured and small constituent nanocrystals can provide a synergistic effect for the enhanced adsorption performance toward As(III) [24]. After being modified on a gold electrode, the Mn doped mesoporous MnFe_2_O_4_ nanocrystal clusters exhibited favorable sensing behavior toward As(III) with the sensitivity of 0.295 μA/ppb and LOD of 1.95 ppb by SWASV [25]. Thus, it is inferred that doping Mn oxides could provide a synergistic effect with Fe and Mn species for As(III) removal or detection contributed to the higher electrochemical reactivity.

In this work, considering the high electrochemical response of Mn doped iron oxide and the excellent electrocatalytical ability of Au nanoparticles towards As(III), we have explored Au nanoparticle decorated mesoporous MnFe_2_O_4_ nanocrystal clusters (MnFe_2_O_4_/Au hybrid nanospheres) for electrochemical sensing of As(III) by square wave anodic stripping voltammetry (SWASV) [26,27]. A glass carbon electrode (GCE) was chosen as the base electrode in order to further reduce the cost. The structure and synergistic effect of the MnFe_2_O_4_/Au hybrid nanospheres were characterized and studied. The optimization of detection conditions—including considerations regarding supporting electrolytes, pH values, deposition potentials, and deposition times—were investigated. Moreover, electrochemical interference behavior, reproducibility, stability, repeatability, and real sample analysis of the MnFe_2_O_4_/Au hybrid nanospheres modified GCE were also studied in detail under the optimum experimental conditions.

## 2. Materials and Methods

### 2.1. Chemical Reagents

All chemicals were analytical grade, and were commercially available from Shanghai Chemical Reagent Co. Ltd without further purification. Acetate buffer solutions of 0.1 M at different pH values were prepared by mixing stock solutions of 0.1 M NaAc, HAc, and NaOH. Phosphate buffer solutions (PBS) of 0.1 M were prepared by mixing stock solutions of 0.1 M H_3_PO_4_, KH_2_PO_4_, K_2_HPO_4_, and NaOH. NH_4_Cl-NH_3_·H_2_O (0.1 M) solutions were prepared by mixing stock solutions of 0.1 MNH_4_Cl and NH_3_·H_2_O in different proportions. All stock and working solutions were prepared in purified water (18.2 MΩ cm), which was obtained through a GWA-UN to Pure & Ultrapure water purification system (Purkinje General).

### 2.2. Apparatus

The Electrochemical measurements were performed on a CHI 660E computer-controlled potentiostat (ChenHua Instruments Co., Shanghai, China). A conventional three-electrode configuration was employed, consisting of a bare or modified glass carbon electrode (GCE) as a working electrode, an Ag/AgCl/saturated KCl electrode as a reference electrode, and a platinum wire as a counter electrode. Scanning electron microscopy (SEM) observation and energy-dispersive X-ray spectrum (EDS) analyses were carried out on a Quanta 200 FEG scanning electron microscope (FEI Company, Hillsboro, OR, USA). The High Resolution Transmission Electron Microscopy (HRTEM) and Transmission Electron Microscopy (TEM) images analyses were carried out on JEM-2010 and JEM-1400 microscopes (JEOL, Tokyo, Japan), respectively.

### 2.3. Preparation of Mesoporous MnFe_2_O_4_/Au Hybrid Nanospheres

Firstly, the mesoporous MnFe_2_O_4_ nanocrystal clusters (MnFe_2_O_4_ NCs (nanocrystal clusters)) were prepared according to a previous report [28]. Then they were APTMS-functionalized according to the previous report with some modification showing as follow [29]: MnFe_2_O_4_ NCs (20 mg) were added to a solution containing ethanol (30 mL) and water (2 mL), followed by the addition of ammonium hydroxide (25%; 2 mL) and APTMS (200 μL). Then, the resulting solution was sonicated continuously for about 8 h. After four-step separation by means of an external magnetic field, the resulting APTMS-functionalized MnFe_2_O_4_ NCs was dissolved in water (10 mL). After being selected with a magnet and rinsed with water four times, the resulting APTMS-functionalized MnFe_2_O_4_ NCs were dissolved in water (10 mL) for further use. 

**Scheme 1:**
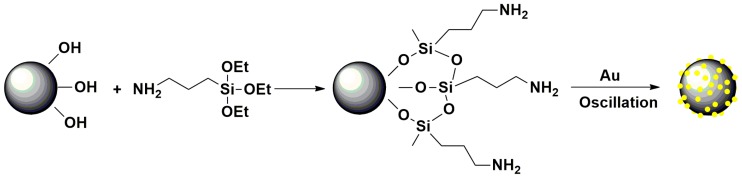
Illustration of the preparation of mesoporous MnFe_2_O_4_/Au hybrid nanospheres.

Subsequently, the solution of APTMS-functionalized MnFe_2_O_4_ NCs (1 mL) was mixed with 20 mL solution of Au nanoparticles via shaking for 24 h. The solution of Au nanoparticles was synthesized according to previously described methods [30]. The resulting product of mesoporous MnFe_2_O_4_/Au hybrid nanospheres was collected through an external magnet. An illustration of the preparation procedure of mesoporous MnFe_2_O_4_/Au hybrid nanospheres can be followed in Scheme 1.

### 2.4. Preparation of Mesoporous MnFe_2_O_4_/Au Hybrid Nanospheres Modified GCE

Firstly, the GCE was polished with 0.3 μm and 0.05 μm alumina power slurries sequentially to form a mirror-shiny surface. Next, it was sonicated with 1:1 HNO_3_ solution, absolute ethanol, and water for 1 min, respectively. Subsequently, the mesoporous MnFe_2_O_4_/Au hybrid nanospheres film on the surface of the GCE was produced as follows: Mesoporous MnFe_2_O_4_/Au hybrid nanospheres (2 mg) were dispersed into purified water (2 mL) to obtain a uniform dispersion by 3 min of sonication. A drop of the above MnFe_2_O_4_/Au hybrid nanospheres solution was pipetted onto the fresh surface of the GCE and dried in air. After evaporation, a thin mesoporous MnFe_2_O_4_/Au hybrid nanospheres film was formed on the surface of the GCE.

### 2.5. Electrochemical Measurements

The electrochemical behavior for the MnFe_2_O_4_/Au hybrid nanospheres modified GCE was observed through square wave anodic stripping voltammetry (SWASV) under the optimized conditions. Under the potential of −0.9 V for 150 s, As(0) was deposited by the reduction of As(III) in 0.1 M HAc-NaAc (pH 5.0). Subsequently, the electrodeposited As(0) was anodic stripped to As(III) which was performed in the potential range of −0.4 to 0.4 V under the following conditions: frequency, 25 Hz; amplitude, 25 mV; increment potential, 4 mV; *vs*. Ag/AgCl. Thereafter, desorption potential of −0.9 V for 150 s was carried out to remove the residual As(0) under stirring conditions. The same experimental conditions were applied in the interference, reproducibility, stability, repeatability, and real sample analysis studies. Cyclic voltammograms (CV) and electrochemical impedance spectra (EIS) were performed in the mixing solution containing 5 mM Fe(CN)_6_^3−/4−^ and 0.1M KCl with the scanning rate of 100 mV∙s^−1^.

## 3. Results and Discussion

### 3.1. Characterization of Mesoporous MnFe_2_O_4_/Au Hybrid Nanospheres

**Figure 1:**
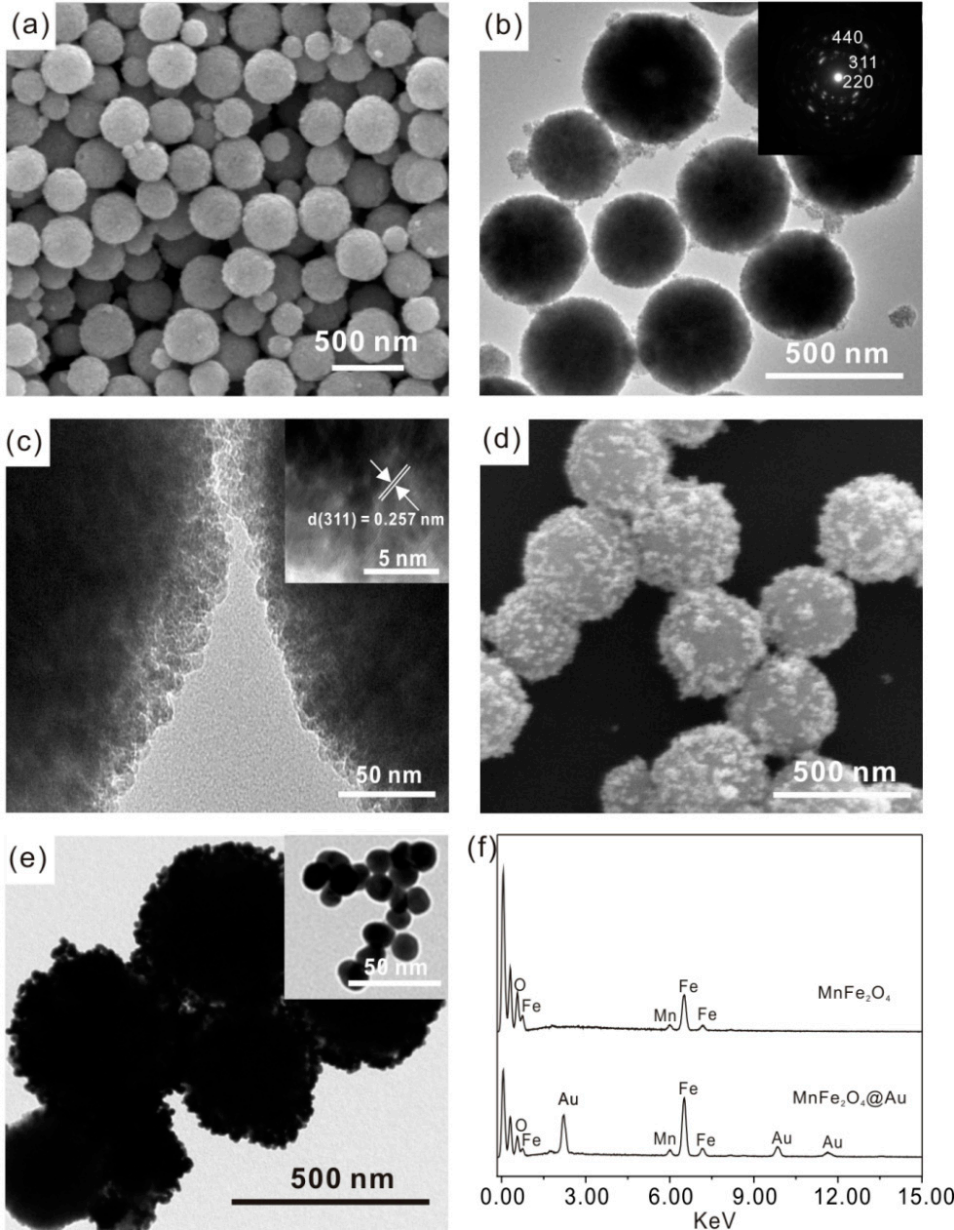
Characterization of mesoporous MnFe_2_O_4_/Au hybrid nanospheres: (**a**) Scanning electron microscopy (SEM) image of MnFe_2_O_4_ NCs; (**b**) and (**c**) The High Resolution Transmission Electron Microscopy (HRTEM) images of MnFe_2_O_4_ NCs, inset in panel b is the corresponding selected-area electron diffraction (SAED) pattern; (**d**) SEM image of MnFe_2_O_4_/Au hybrid nanospheres; (**e**) TEM image of MnFe_2_O_4_/Au hybrid nanospheres, inset in panel e is the TEM image of Au nanoparticle decorated on the surface of MnFe_2_O_4_ NCs; (**f**) Energy-dispersive X-ray spectrum (EDS) of MnFe_2_O_4_ NCs and MnFe_2_O_4_/Au hybrid nanospheres.

Figure 1a–c show the as-prepared mesoporous MnFe_2_O_4_ sample is made up of monodisperse, uniform, and smooth-faced microspheres with diameters of about 350 nm. The HRTEM image indicates that the individual microsphere contains lots of loose clusters, which are composed of primary nanocrystals with the size of about 8–12 nm (Figure 1b). This mesoporous architecture might provide the microspheres with large surface areas in favor of adsorbing guest molecules [31,32,33]. The selected-area electron diffraction (SAED) pattern indicates that the diffraction spots are widened into narrow arcs (insets of Figure 1b), also signifying the clusters are composed of many misaligned ferrite nanocrystals [31,34]. The d value of the crystal plane (311) shown in Figure 1c verifies the crystalline structure of MnFe_2_O_4_ NCs. Taking the analyses of SEM and HRTEM mentioned above, MnFe_2_O_4_ NCs with diameters of 350 nm and mesoporous structures composed of nanocrystals with the size of about 11 nm have been prepared successfully, which might render them with large surface areas in favor of adsorbing and detecting heavy metal ions. Figure 1d,e show the SEM and TEM images of mesoporous MnFe_2_O_4_/Au hybrid nanospheres. The corresponding images show that 13 nm Au nanoparticles (insets of Figure 1e) are decorated on the surface of mesoporous MnFe_2_O_4_ microspheres. The hybrid nanospheres with rough surfaces decorated by Au nanoparticles are able to combine the good catalytic activity of Au and high adsorption activity of mesoporous MnFe_2_O_4_ toward heavy metal ions simultaneously, thus it might offer good electrochemical activity for detection toward trace As(III).

### 3.2. Electrochemical Characterization of Mesoporous MnFe_2_O_4_/Au Hybrid Nanospheres Modified GCE

**Figure 2:**
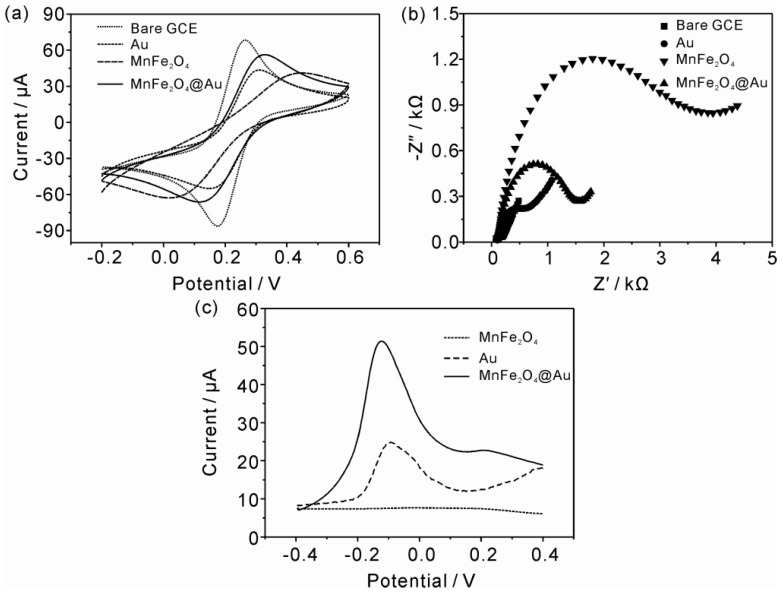
Cyclic voltammograms (**a**) and Nyquist diagram of electrochemical impedance spectra (**b**) for bare glass carbon electrode (GCE), Au nanoparticles (NPs), MnFe_2_O_4_ NCs, MnFe_2_O_4_/Au hybrid nanospheres modified GCE in the solution of 5 mM Fe(CN)_6_^3−/4−^ containing 0.1 M KCl. (**c**) SWASVs (square wave anodic stripping voltammetries) for 50 ppb As(III) on the MnFe_2_O_4_ NCs, Au NPs, MnFe_2_O_4_/Au hybrid nanospheres modified GCE in 0.1 M HAc-NaAc (pH 5.0). Deposition potential: −0.9 V, deposition time: 150 s, step potential: 4 mV, amplitude: 25 mV, frequency: 25 Hz, *vs.* Ag/AgCl.

The typical cyclic voltammetry (CV) of bare GCE, Au, MnFe_2_O_4_ NCs, and MnFe_2_O_4_/Au hybrid nanospheres modified GCE were electrochemically characterized in a solution of 5 mM Fe(CN)_6_^3−/4−^ with 0.1 M KCl (Figure 2a). Both the bare and modified GCE were observed to exhibit a pair of well-defined reversible redox peaks. Compared with the bare GCE, Figure 2a displays the anodic and cathodic peak currents for Au, MnFe_2_O_4_ NCs, and MnFe_2_O_4_/Au hybrid nanospheres modified GCE which all decline apparently, which thereby indicates that the bare GCE has been modified successfully by the above nanomaterials. Among all the modified GCE, it can be found that the anodic and cathodic peak currents of the MnFe_2_O_4_ NCs modified GCE is the lowest, which is due to the poor conductivity of the metal oxide on the surface of the bare GCE which can slow the electron transfer [35]. However, after being decorated by Au nanoparticles, the anodic and cathodic peak currents of MnFe_2_O_4_/Au hybrid nanospheres modified GCE observably increases and the peak-to-peak separation (△Ep) decreases from 190 mV to 120 mV, indicating the electron transfer of MnFe_2_O_4_ NCs were improved observably by the hybrid of the Au nanoparticles on the surface of MnFe_2_O_4_ NCs.

The interface properties of the bare GCE, Au, MnFe_2_O_4_ NCs, and MnFe_2_O_4_/Au hybrid nanospheres modified GCE are further analyzed by electrochemical impedance spectroscopy (EIS). The diameter of the semicircle equals the electron transfer resistance (R_et_), and the R_et_ controls the electron transfer kinetics of the redox probe at the surface of the electrode. Figure 2b shows the EIS of bare GCE displays an almost straight line, implying the bare GCE conducts electricity very well. In addition, there is a small semicircle domain for the EIS of Au nanoparticles modified GCE, thereby meaning a very low R_et_ for this modified electrode. However, Figure 2b shows the R_et_ of the MnFe_2_O_4_ NCs modified GCE was much higher than the bare or Au nanoparticles modified GCE, indicating that the MnFe_2_O_4_ NCs layer blocked the electron transfer of the electrochemical probe. Due to the poor conductivity of metal oxide, the modification of MnFe_2_O_4_ NCs could further pose a barrier and hinder the access of the redox probe to the electrode surface, thus resulting in a large R_et_ of the MnFe_2_O_4_ NCs modified GCE. Interestingly, the EIS of the MnFe_2_O_4_/Au hybrid nanospheres modified GCE shows a much smaller semicircle compared with the MnFe_2_O_4_ NCs modified GCE, indicating that the decorating of Au nanoparticles on the surface of MnFe_2_O_4_ NCs could improve the electron transfer of MnFe_2_O_4_ NCs on the surface of the GCE, which is consistent with the above CV results.

Figure 2c presents the SWASV analytical results of the MnFe_2_O_4_ NCs, Au nanoparticles, and MnFe_2_O_4_/Au hybrid nanospheres modified GCE. When the accumulation process was performed for 150 s at −0.9 V in 50 ppb As(III) solution containing 0.1 M acetate buffer (pH 5.0), it shows no peak appears in the curve of the MnFe_2_O_4_ NCs modified GCE, and a weak peak at −0.9 V can be observed for the Au nanoparticles modified GCE. For the MnFe_2_O_4_/Au hybrid nanospheres modified GCE, the peak current increases much higher than the single MnFe_2_O_4_ NCs or Au nanoparticles modified GCE. It shows that the hybrid that consists of Au nanoparticles on the surface of MnFe_2_O_4_ NCs could provide much more remarkable electrochemical performances towards As(III) detection. It might be attributed to the catalytic effect and high electron transfer of Au nanoparticles would provide much more active sites for the electrochemical reactions in the detection process of As(III). Thus, based on the unique mesoporous structure of the MnFe_2_O_4_ NCs responses to As(III) [25], the hybrid of Au nanoparticles on the surface of MnFe_2_O_4_ NCs indeed provides a synergistic effect for the detection of As(III) through SWASV.

### 3.3. Optimum Experimental Conditions of Electrochemical Detection of As(III) 

**Figure 3:**
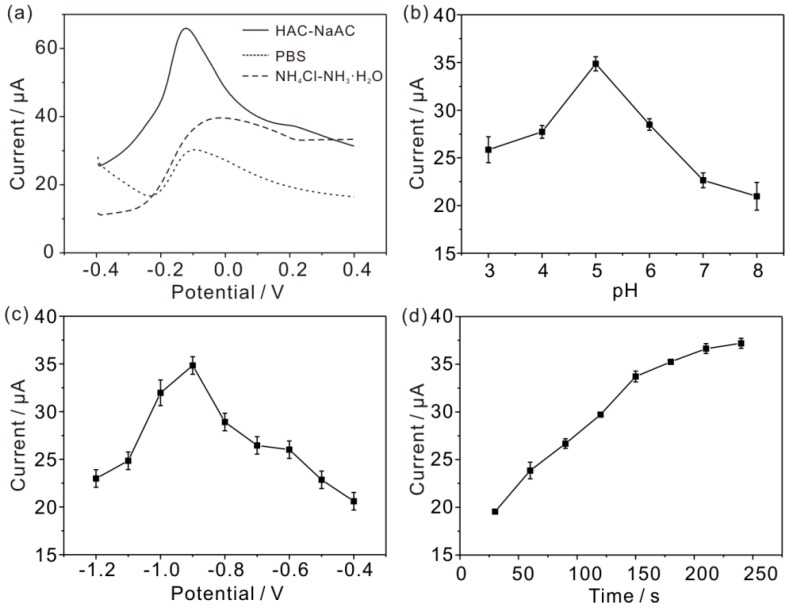
Influence of (**a**) supporting electrolytes; (**b**) pH value; (**c**) deposition potential; and (**d**) deposition time on the voltammetric response of the MnFe_2_O_4_/Au hybrid nanospheres modified GCE. Data were evaluated by SWASV of 50 ppb As(III).

The optimization of experimental conditions (supporting electrolytes, pH, deposition potential, and deposition time) was taken into account using SWASV. The effect of supporting electrolytes at pH 5.0 was tested and NaAc-HAc was finally chosen as the best electrolyte in comparison with PBS and NH_4_Cl-NH_4_OH (Figure 3a). The effect of pH on the SWASV response was investigated in the pH range of 3.0–8.0 in 0.1 M NaAc-HAc solutions, as shown in Figure 3b. The maximum peak current was achieved when the solution was maintained at pH 5.0. The decline of the pH value would provide a more acidic environment resulting in the destruction of mesoporous MnFe_2_O_4_ NCs, thus the stripping current became poor. The stripping current also observably declined when the pH value increased to a high level, such as 7 or 8, which is consistent with the report that the adsorption capacity of As(III) decreased under higher pH values [36]. In a higher pH solution, the electrostatic attraction will weaken between the negatively charged As(III) species and positively charged surface sites of MnFe_2_O_4_ NCs. Thus, pH 5.0 was chosen for the preconcentration solution in this experiment. Different deposition potentials in the range of −1.2 to −0.4 V were also experimented with via SWASV by standard additions of 50 ppb As(III) in 0.1 M NaAc-HAc (pH 5.0), as shown in Figure 3c. It was found that the deposition potential negative shifts from −0.4 V to −0.9 V (*vs*. Ag/AgCl) can obviously improve the peak current of reduction of As(III). Generally, the peak current would reach a plateau, settling to a constant value which should be the optimum deposition potential. However, when the potentials beyond −0.9 V were applied, a decrease response of the peak current were observed, as shown in Figure 3c, due to the reduction of hydrogen, which would lead to the formation of micro gas bubbles and thus reduce the effective electrode area and weaken the response of As(III) [35]. Therefore, the deposition potential of −0.9 V was selected for further studies. The dependence of the stripping peak current of As(III) at the deposition time on the sensing response was studied under 50 ppb As(III) (pH 5.0). Figure 3d shows the stripping peak current improved with the deposition time increase from 30 s to 240 s. Although the sensitivity was improved under a longer deposition time, it also reduced the upper detection limit due to the surface saturation in the high metal ion concentration [37]. When the deposition time exceeded 150 s, the increased velocity of the stripping current become slower, suggesting that the electrode surface was approximately saturated by the As(III). Considering the time consumed, the optimized deposition time of 150 s was used throughout. At last, the optimum experimental conditions for the electrochemical detection of As(III) was determined in 0.1 M NaAc-HAc (pH 5.0) by depositing it for 150 s under the deposition potential of −0.9 V.

### 3.4. Electrochemical Detection of As(III) with MnFe_2_O_4_/Au Hybrid Nanospheres Modified GCE

**Figure 4:**
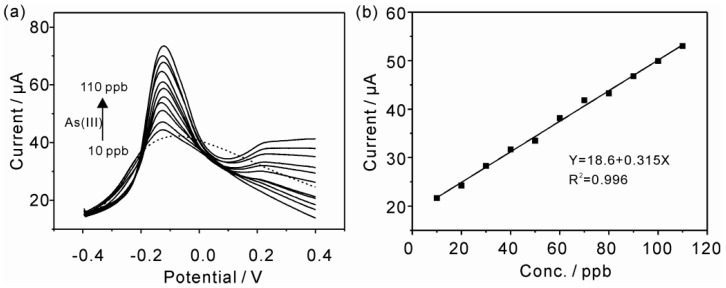
SWASV responses (**a**) and the corresponding calibration plot (**b**) of the MnFe_2_O_4_/Au hybrid nanospheres modified GCE toward As(III) over a concentration range of 10 to 110 ppb by depositing for 150 s in 0.1 M HAc-NaAc (pH 5.0). Deposition potential: −0.9V, step potential: 4 mV, amplitude: 25 mV, and frequency: 25 Hz.

Under the optimal experimental conditions, As(III) was determined on the MnFe_2_O_4_/Au hybrid nanospheres modified GCE using SWASV. Figure 4a presents the SWASV response toward As(III) over the concentration ranges of 10 to 110 ppb. The linearization equation was i/μA = 18.6 + 0.315 c/ppb, with the correlation coefficient of 0.996 (Figure 4b). Based on a signal-to-noise ratio equal to 3 (3σ method), the theoretical limit of detection (LOD) was estimated to be 3.37 ppb. The sensitivity and LOD of this study were compared with other previously reported electrodes listed in Table 1. In contrast to other Au electrode systems, it can be observed that the proposed electrode can obtain preferable sensitivity (0.315 μA/ppb) while remaining cheap at the same time. Although the LOD of 3.37 ppb is high, it is still within the maximum permissible limits for As(III) in drinking water issued by the World Health Organization (10 ppb). However, compared with the Fe_3_O_4_-RTIL modified SPCE [18], the sensitivity and LOD of this proposed sensor was at a disadvantage. It was considered that the Fe_3_O_4_-RTIL composite could provide a specific interface for arsenic to accumulate and exchange electrons, thus future studies focusing on the MnFe_2_O_4_-Au-RTIL composite may obtain exciting sensitivity and LOD for the electrochemical sensing of As(III).

### 3.5. Interference Measurements

**Figure 5:**
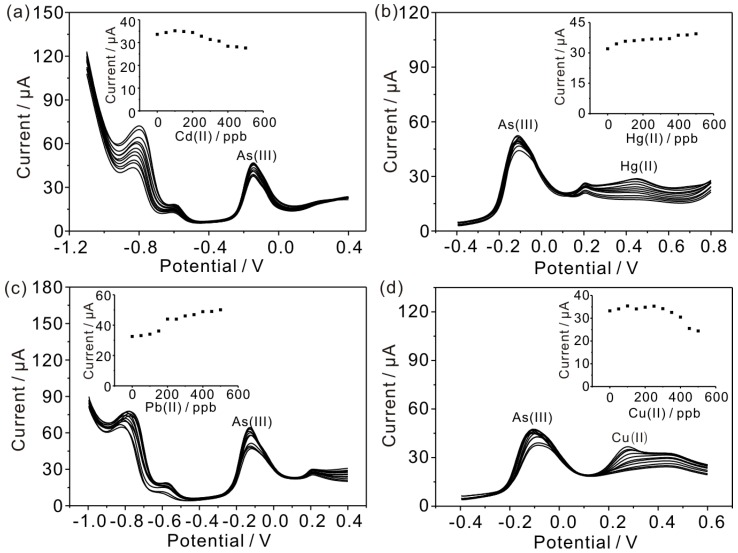
SWASV responses of the MnFe_2_O_4_/Au hybrid nanospheres modified GCE containing 50 ppb As(III) in the presence of Cd(II), Hg(II), Pb(II), and Cu(II) over a concentration range of 0 to 500 ppb, respectively. (Insets in panel a, b, c, and d are the corresponding peak currents toward 50 ppb As(III) over a concentration range of 0 to 500 ppb of interfering ions.) Electrochemical stripping conditions are identical to those in Figure 4.

Commonly, the other metal ions can coprecipitate and strip off under the experimental conditions for determination of As(III), thus sensitive detection of As(III) in the real sample without interference is a challenging task. In order to investigate the interference of other heavy metal ions to the electrochemical determination of As(III), a series of interference measurements were studied. Figure 5 presents the SWASV response of MnFe_2_O_4_/Au hybrid nanospheres modified GCE in 0.1 M HAc-NaAc (pH 5.0) containing 50 ppb As(III) in the presence of Cd(II), Hg(II), Pb(II), and Cu(II) and over a concentration range of 0 to 500 ppb, respectively. Figure 5a,b show the peak stripping currents of As(III) almost stays the same as the concentrations of Cd(II) and Hg(II) increase linearly. When the concentrations of Cd(II) and Hg(II) are up to 10-fold the concentration of As(III), the peak current of As(III) still changes a little. It means Cd(II) and Hg(II) do not observably interfere with the detection of As(III) for the MnFe_2_O_4_/Au hybrid nanospheres modified GCE when sensing the two target metal ions simultaneously. As to the interference of Cu(II), Figure 5c indicates the peak currents of As(III) also remain the same when the concentration of Cu(II) is less than 300 ppb. However, the peak current of As(III) drops observably when the concentration of Cu(II) increases further. Therefore, it means that Cu(II) would interfere with the detection of As(III) for the MnFe_2_O_4_/Au hybrid nanospheres modified GCE when the concentration of Cu(II) is beyond a certain value when the Cu(II) and As(III) coexist within a solution. On the other hand, Figure 5d shows that the peak current of As(III) almost rises linearly as the concentration of Pb(II) increases from 0 to 500 ppb. These results indicate that the existence of Pb(II) would enhance the detection signal of As(III) to some extent. This can be reasoned by the mutual promotion of adsorption sites between Pb(II) and As(III) before the adsorbing capacity of heavy metal ions reaches saturation.

### 3.6. Reproducibility, Stability and Repeatability

**Figure 6:**
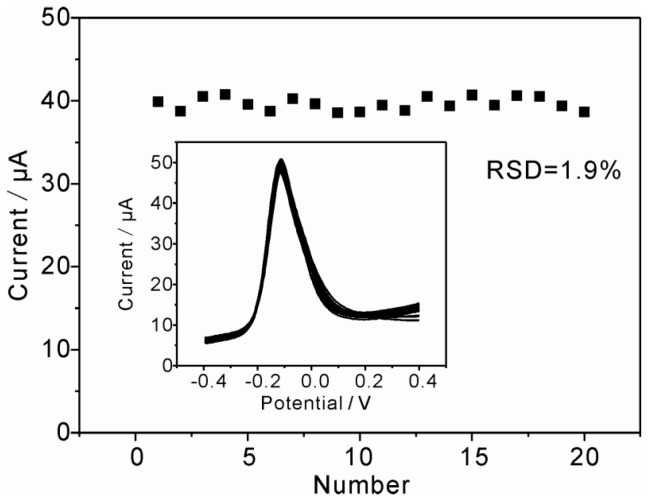
Repeatability measurements of 50 ppb As(III) on MnFe_2_O_4_/Au hybrid nanospheres modified GCE towards As(III) in 0.1 M HAc-NaAc (pH 5.0). Data are collected from every SWASV response shown in the inset. Experimental conditions are identical to those in Figure 4.

To evaluate the reproducibility of the modified electrode, six MnFe_2_O_4_/Au hybrid nanospheres modified GCEs were prepared following the same procedure and were applied to detect 50 ppb As(III) in 0.1 M HAc-NaAc (pH 5.0) under the optimized conditions. The relative standard deviation (RSD) derived from the peak currents from six tests was 3.9%, indicating that the developed method has good reproducibility. Additionally, the stability of the modified electrodes were investigated by storing six prepared electrode at 4 °C for ten days, and then testing the SWASV response toward 50 ppb As(III) under the optimized conditions. The result showed that the stripping peak current only decreased by 7.8%, 7.3%, 3.7%, 4.6%, 7.0%, and 4.8% of the first value, thereby showing a long-term stability of the fabricated electrode. Moreover, the repeatability of the MnFe_2_O_4_/Au hybrid nanospheres modified GCE were also evaluated by conducting repetitive experiments for 20 times to detect 50 ppb As(III) on the modified electrode under the optimized conditions. Figure 6 shows the stripping current was nearly constant after continuous cycling for 20 times, and no obvious changes in the peak currents were observed with the RSD of 1.9%. The good reproducibility, long-term stability, and favorable repeatability of MnFe_2_O_4_/Au hybrid nanospheres modified GCE make them attractive for the preparation of a electrochemical sensor toward As(III).

### 3.7. Real Sample Analysis

**Figure 7:**
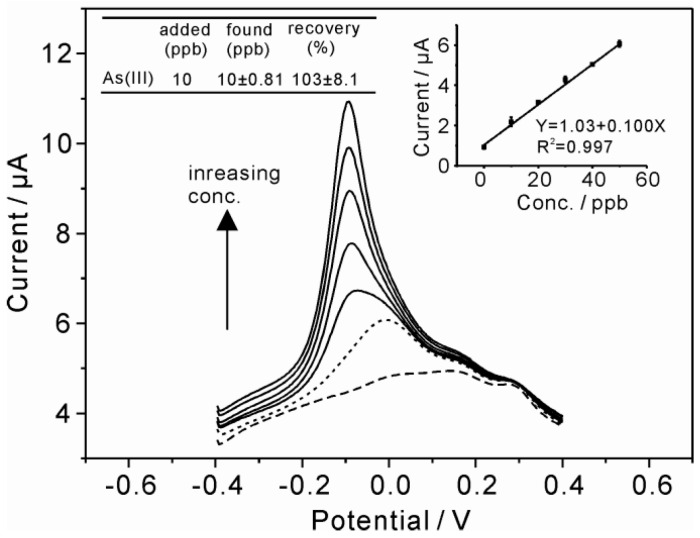
SWASV responses of standard additions of As(II) into a real water sample diluted with 0.1 M NaAc-HAc solution (pH 5.0) in a ratio of 1:9 and the corresponding calibration plot of peak current against As(II) concentrations. The dash dotted line is the baseline in the solution of 0.1 M NaAc-HAc (pH 5.0) containing 1.0 mL of the real sample. The dotted line is the SWASV response toward 10 ppb As(III) in the solution of 0.1 M NaAc-HAc (pH 5.0) containing 1.0 mL of the real sample. The inset table is the recovery calculation. Error bars are the standard deviation for three consecutive measurements. SWASV conditions are identical to those in Figure 4.

In order to evaluate the practical application of the MnFe_2_O_4_/Au hybrid nanospheres modified GCE, real water sample analyses were taken from laboratory tap water [26,42]. The real sample was diluted with a 0.1 M HAc-NaAc buffer solution (pH 5.0) in a ratio of 1:9 without any further treatment. The standard addition of 10 ppb As(III) was performed in the diluted sample and the recovery studies were carried out by further standard additions of As(III) into the diluted sample with a known concentration of As(III). The SWASV response and the corresponding calibration plot of peak currents against As(III) concentrations are presented in Figure 7. No obvious signals for As(III) were observed in the real samples (dash dotted line in Figure 7), indicating no As(III) was detected in the laboratory tap water. Furthermore, the recovery obtained is calculated to be 103% ± 8.1%, which reveals that the proposed detection method for As(III) has the potential for practical application.

## 4. Conclusions

In summary, Au nanoparticles decorated mesoporous MnFe_2_O_4_ nanocrystal clusters (MnFe_2_O_4_/Au hybrid nanospheres) with high electrochemical performance were used for the electrochemical determination of As(III) by square wave anodic stripping voltammetry (SWASV). The as-prepared product was characterized by SEM, HRTEM, TEM, and EDS. The results suggest that 13 nm Au nanoparticles were indeed supported on the surface of mesoporous MnFe_2_O_4_ microspheres with the diameter of about 350 nm. Electrochemical behavior of the MnFe_2_O_4_/Au hybrid nanospheres modified GCE toward As(III) showed favorable sensitivity (0.315 μA/ppb) and LOD (3.37 ppb) for As(III) under the optimized conditions in 0.1 M NaAc-HAc (pH 5.0) by depositing for 150 s at the deposition potential of −0.9 V. No obvious interference from Cd(II) and Hg(II) was recognized during the detection of As(III). In addition, the excellent reproducibility, stability, and repeatability of MnFe_2_O_4_/Au hybrid nanospheres made it a promising electrode material for the electrochemical determination of As(III). Furthermore, the MnFe_2_O_4_/Au hybrid nanospheres modified GCE offered potential practical applicability in the electrochemical detection of As(III) in real water samples. Ultimately, the present work may provide a potential method for the design of new and cheap sensors in the application of electrochemical detection toward trace As(III) and other toxic metal ions.

## Figures and Tables

**Table 1:** Comparison of current sensitivity with previously reported values of different electrodes for electrochemical detection of As(III).

Electrode	Sensitivity (μA/ppb)	LOD (ppb)	Refs.
Au-UMEA	0.044	0.013	[38]
Au NPs/GCE	0.24	0.0096	[3]
PBSPE	0.000387	1.875	[39]
Gold-carbon composite electrode	0.133	0.375	[40]
MWCNTs/gold electrode	0.236	-	[41]
Co_3_Ox/GCE	0.00148	0.825	[17]
MnFe_2_O_4_/gold electrode	0.295	1.95	[25]
MnFe_2_O_4_/Au modified GCE	0.315	3.37	This work
Fe_3_O_4_-RTIL modified SPCE	4.91	8 × 10^−4^	[18]
